# Evolution of the conductive filament system in HfO_2_-based memristors observed by direct atomic-scale imaging

**DOI:** 10.1038/s41467-021-27575-z

**Published:** 2021-12-13

**Authors:** Ying Zhang, Ge-Qi Mao, Xiaolong Zhao, Yu Li, Meiyun Zhang, Zuheng Wu, Wei Wu, Huajun Sun, Yizhong Guo, Lihua Wang, Xumeng Zhang, Qi Liu, Hangbing Lv, Kan-Hao Xue, Guangwei Xu, Xiangshui Miao, Shibing Long, Ming Liu

**Affiliations:** 1 Key Laboratory of Microelectronic Devices & Integration Technology, Institute of Microelectronics of Chinese Academy of Sciences, Beijing, 100029 China; 2 School of Microelectronics, University of Science and Technology of China, Hefei, 230026 China; 3 University of Chinese Academy of Sciences, Beijing, 100049 China; 4 School of Integrated Circuits, School of Optical and Electronic Information, Huazhong University of Science and Technology, Wuhan, 430074 China; 5 Institute of Microstructure and Property of Advanced Materials, Beijing Key Laboratory of Microstructure and Property of Advanced Materials, Beijing University of Technology, Beijing, 100124 China; 6 Frontier Institute of Chip and System, Fudan University, Shanghai, 200433 China

**Keywords:** Nanoscale materials, Electronic devices

## Abstract

The resistive switching effect in memristors typically stems from the formation and rupture of localized conductive filament paths, and HfO_2_ has been accepted as one of the most promising resistive switching materials. However, the dynamic changes in the resistive switching process, including the composition and structure of conductive filaments, and especially the evolution of conductive filament surroundings, remain controversial in HfO_2_-based memristors. Here, the conductive filament system in the amorphous HfO_2_-based memristors with various top electrodes is revealed to be with a quasi-core-shell structure consisting of metallic hexagonal-Hf_6_O and its crystalline surroundings (monoclinic or tetragonal HfO_x_). The phase of the HfO_x_ shell varies with the oxygen reservation capability of the top electrode. According to extensive high-resolution transmission electron microscopy observations and ab initio calculations, the phase transition of the conductive filament shell between monoclinic and tetragonal HfO_2_ is proposed to depend on the comprehensive effects of Joule heat from the conductive filament current and the concentration of oxygen vacancies. The quasi-core-shell conductive filament system with an intrinsic barrier, which prohibits conductive filament oxidation, ensures the extreme scalability of resistive switching memristors. This study renovates the understanding of the conductive filament evolution in HfO_2_-based memristors and provides potential inspirations to improve oxide memristors for nonvolatile storage-class memory applications.

## Abstract (web summary)

Understanding the mechanism of the formation and rupture of conductive filaments in HfO_2_-based memristors is essential to solve the problem of scalability of the devices. Here, Zhang et al. visualize this process by tracking atomic-scale evolution of conductive filaments during resistive switching cycles.

## Introduction

Developing memory devices based on novel operation principles, innovative structures, and new materials is a fundamental and inevitable solution to acquire faster and denser nonvolatile memory (NVM) to meet the requirements of big data, cloud computing, artificial intelligence, and new industries in the modern information society^1–5^. Compared with the current mainstream charge-based flash memory, resistive switching random access memory (RRAM or so-called memristor), as one of the most promising candidates for next-generation NVM, has the advantages of high-speed operation, a scalable two-terminal structure for high-density 3D integration, excellent compatibility with the back-end of the traditional CMOS process, and analog characteristics for novel in-memory computing^6–10^. Resistive switching (RS) behavior stemming from the repeatable formation/rupture of conductive filaments (CFs) under an external electric field lays the foundation of oxide memristors^11,12^. The formation and rupture of CFs yield the low resistance state (LRS) and high resistance state (HRS) of the oxide memristor, respectively. The physical mechanism of filamentary switching includes the generation, migration, and recombination of defects such as oxygen vacancies or metal ions in the RS layer^13–15^. These processes will result in variations in the chemical composition and structure of the local switching region, i.e., the CF region within the RS layer^16–18^.

Binary oxide materials, including ZrO_2_^19^, TiO_2_^20^, TaO_x_^21,22^, ZnO^23^, NiO^24^, CuO_x_^25^, etc., have been widely investigated in memristors on account of their simple composition, modulation convenience, excellent scalability, and CMOS process compatibility^26–28^. Various investigations have been separately carried out on the microscopic properties of CFs in memristors based on the oxides above, including the morphology, size, quantity, chemical composition, crystal structure, and dynamic growth/rupture process^29–31^. As a well-known high-k oxide dielectric material, hafnia (HfO_2_) has attracted considerable interests and is widely recognized as one of the most promising CMOS-compatible RS materials^32,33^. Similar to the situation in memristors based on other oxides, CFs in the HfO_2_-based valence change mechanism (VCM) or thermochemical mechanism (TCM) memristors have also been demonstrated as a low-oxygen content region^34,35^. Through high-resolution transmission electron microscopy (HRTEM) observation^36^, CFs were identified to be the directionally aligned crystalline regions in amorphous HfO_2_ (a-HfO_2_) consisting of monoclinic and orthorhombic oxygen-deficient phases. Notably, the core–shell structure (oxygen-deficient CF and the corresponding oxygen-rich shell) was predicted by ab initio calculations combined with experimental studies for Pt/HfO_2_/Pt memristors^37^. In addition, ref. ^38^ reported the existence of a low-conductivity region with excess oxygen around the oxygen-deficient (or oxygen-vacancy-rich) CF in a Pt/Hf/HfO_2_/Pt memristor based on synchrotron-based scanning transmission X-ray microscopy analysis.

The abovementioned studies indicate that the formation/rupture of electric field-induced oxygen-deficient CFs with relatively high conductance contributes to the RS behavior, and this scenario dominates the physical understanding of HfO_2_-based memristors. However, the dynamic changes in the physical properties of the CF system (CFs and their surroundings) in the RS process, including the composition, structure, and especially the evolution of CF surroundings in HfO_2_-based RS memristors, are generally less focused on in previous studies and lack direct observation at the atomic scale. More detailed knowledge about the physical properties of dynamic CF systems and the RS mechanism is essential for the development of the large-scale manufacturing and commercialization of HfO_2_-based RS memory.

In this work, we investigate the dynamic evolution characteristics of the CF system specifically in a Pt/HfO_2_/Pt crossbar RS memristor. The inert Pt electrode, which scarcely participates in the RS process, renders the Pt/HfO_2_/Pt structure a pure system to study the structure and chemical composition of the oxygen-deficient CFs in oxide memristor devices. The devices show considerable RS performance, including a large switching window (HRS/LRS) above 10^6^ and a short OFF/ON switching time within 120/20 ns. The atomic structure of the oxygen-deficient CF is clearly revealed to be crystalline hexagonal-Hf_6_O (h-Hf_6_O) for the first time through HRTEM. The hexagonal-crystal Hf_6_O can be viewed as the standard hexagonal metal Hf with one interstitial oxygen atom in the Hf_6_ ring. Interestingly, the CF system here is based on a quasi-core–shell structure, since both nonconductive monoclinic and tetragonal HfO_2_ (m-HfO_2_ and t-HfO_2_) are observed to surround the complete and ruptured h-Hf_6_O CFs, respectively, in the Pt/HfO_2_/Pt memristor. Therefore, the RS process of HfO_2_-based memristors can be accompanied by the transition between distinct crystalline phases, owing to the Joule heating effect and variation of oxygen vacancy concentration. Ab initio calculations were conducted to reveal the energetics of the HfO_2_ phase transition and filamentary conduction, and the results were in good agreement with the experimental data. HfO_2_-based RS memristors with application-oriented electrodes (TiN, Ta, Hf, and Ti) yield CF systems quite similar to those of Pt/HfO_2_/Pt devices, suggesting the universal significance of the quasi-core–shell CF structure. This study provides a further understanding of the nature of the CF system and supplies submechanisms towards the VCM and TCM of HfO_2_-based memristors. The core-shell nature of the CF system in HfO_2_-based memristors may renew the interests in the RS mechanism study.

## Results

### Device fabrication and switching performance

**Fig. 1:**
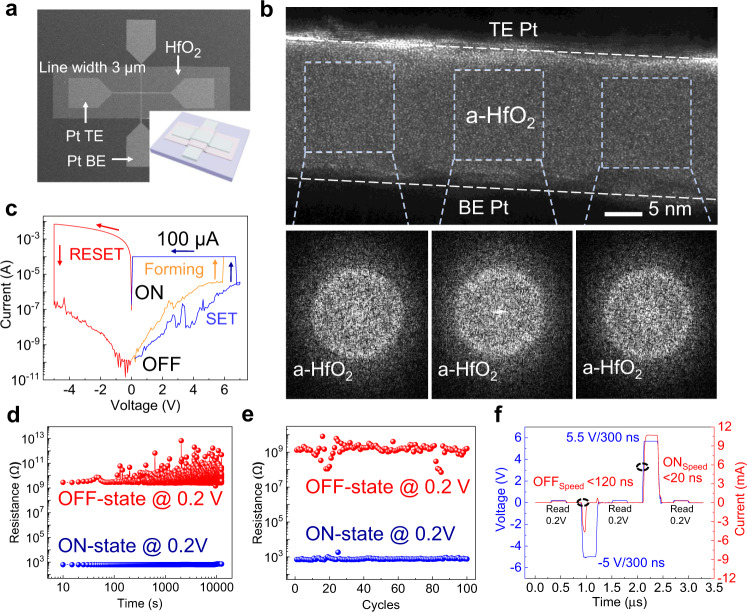
Structure and RS performance of the Pt/HfO_2_/Pt memristor. **a** SEM image and schematic illustration of the Pt/HfO_2_/Pt memristor. The effective line width of the memristor is 3 μm. **b** HRTEM image of the fresh Pt/HfO_2_/Pt stack and the typical FFT diffraction patterns of the marked region of the HfO_2_ RS layer, indicating the amorphous morphology of the as-fabricated HfO_2_ film. **c** Typical RS *I*–*V* characteristics of the device. **d** Retention characteristics of the HRS and LRS of the device for 10^4^ s. **e** The resistance distribution of HRS and LRS of 100 switching cycles from the 10 randomly selected devices. **f** Typical *V*-*t* and *I*–*t* synchronous curves of the OFF and ON switching processes under the pulse mode, where both the LRS and the HRS can be reversed to their opposites within a 300 ns write or erase pulse.

To investigate the evolution of the CF structure and its surroundings in the HfO_2_ RS layer, Pt/HfO_2_/Pt crossbar RS memristors were fabricated. The detailed fabrication process is elaborated in Methods section and Supplementary Fig. 1. As seen from the scanning electron microscopy (SEM) image in Fig. 1a, the effective area of the crossbar cell is 3 × 3 µm^2^. A schematic illustration of the device is displayed in the inset, and the thicknesses of the bottom electrode (BE), RS layer, and top electrode (TE) are 40, 20, and 30 nm, respectively. As demonstrated in Fig. 1b, the as-deposited HfO_2_ RS layer from the fresh Pt/HfO_2_/Pt stack contains a-HfO_2_ throughout the device region, as evidenced by the HRTEM image and fast Fourier transform (FFT) diffraction patterns of the marked regions.

The Pt/HfO_2_/Pt memristors were operated under typical bidirectional nonvolatile RS mode with a 100 μA compliance current (*I*_CC_). The *I*_CC_ current limit could protect the device from hard breakdown during electrical operations. For all electrical measurements, the TE was biased, while the BE was grounded. Figure 1c presents typical Forming (orange line), RESET (red line), and SET (blue line) *I*–*V* curves of the Pt/HfO_2_/Pt memristor. The Forming process is generally utilized to initialize the formation of CFs and subsequently trigger repeatable CF rupture/connection behavior under the RESET/SET biases, which dominates the OFF/ON behavior of oxide memristors^39^. Based on the statistical analysis on SET and Forming voltages (*V*_SET_ and *V*_Forming_) in HfO_2_-based memristors with Pt, TiN, Ti, Hf, and Ta TEs, as shown in Supplementary Fig. 2, the situation that *V*_SET_ is higher than *V*_Forming_, corresponds to a probability event, which tends to occur in HfO_2_-based memristors with strong oxygen-reservation electrodes (e.g., Ti, Hf, Ta). At a read bias of 0.2 V, the switching window of the HRS/LRS ratio can be as high as 10^6^. As confirmed by temperature-resistance electrical tests (Supplementary Fig. 3), the LRS and the HRS are identified to have metallic and semiconductive characteristics, respectively. From the retention measurement (read at 0.2 V) shown in Fig. 1d, the HRS, LRS, and HRS/LRS ratio are well maintained for 10^4^ s without obvious degradation. The HRS fluctuation can be attributed to random noise and current undulation when approaching the test limit of the measurement system. In addition, the 10 stochastically selected devices all maintain an HRS/LRS ratio above 10^4^ during 100 switching cycles, as shown in Fig. 1e, indicating the considerable uniformity of the Pt/HfO_2_/Pt RS memristors. The switching time of the device was evaluated by *V*–*t* and *I*–*t* synchronous curves measured under pulse mode, as shown in Fig. 1f, where both the LRS and the HRS switch to their opposites under a 300 ns negative RESET or positive SET pulse. As marked by the black dashed circles, the OFF and ON times are determined to be within 120 ns and 20 ns, respectively.

### HRTEM observation of the CF system

**Fig. 2:**
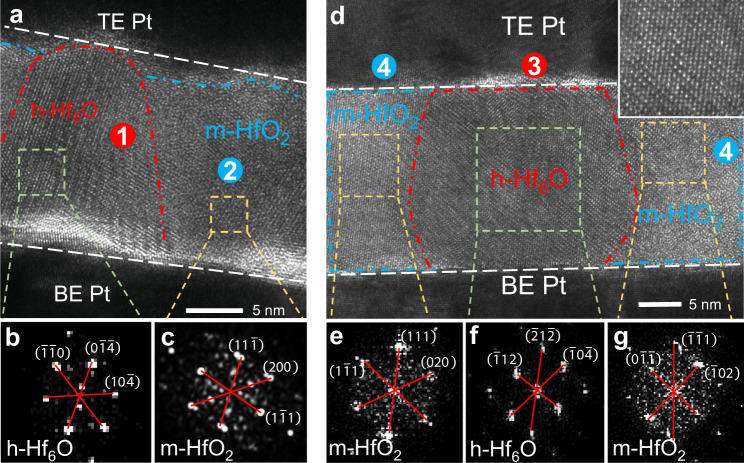
Complete CFs and their m-HfO_2_ shells in the LRS of Pt/HfO_2_/Pt RS memristors. **a** HRTEM of a complete CF in the LRS device operated under 0.1 mA *I*_CC_ with the typical polymorphous HfO_x_ region, namely, h-Hf_6_O and m-HfO_2_ region, as confirmed by their FFT diffraction patterns in **b** and **c**. **d** HRTEM of the CF in the LRS memristor operated under 1 mA *I*_CC_, where the h-Hf_6_O CF is enclosed by the nonconductive m-HfO_2_ shell, as confirmed by their FFT diffraction patterns in **e**–**g**. The inset of **d** is the high-resolution close-up view of the h-Hf_6_O CF region marked with a green square.

HRTEM helps to observe any tiny changes inside the RS layer to reveal the RS mechanism and the CF nature at the atomic scale^34,36,40^. The current, which flows through the memristor, greatly influences the morphology of the CF by generating Joule heat. The typical changes in the RS layer structure of the LRS Pt/HfO_2_/Pt memristor device induced by the SET behavior under 0.1 mA and 1 mA *I*_CC_ are captured in the HRTEM images in Fig. 2. Different from the amorphous nature of the as-deposited HfO_2_ layer, a clear crystal lattice can be observed after the SET operation, as outlined by the red and blue arc curves in Fig. 2a (*I*_CC_ = 0.1 mA) and 2d (*I*_CC_ = 1 mA). Note that the crystallization of local regions in the amorphous RS layer are expected to be driven by the Joule heat effect of the nearby CF current^31,41^. Therefore, we looked for possible CFs near the crystallization regions in the RS layer. According to different crystal categories, both TEM images can be divided into two typical regions. The FFT diffraction pattern in region 1 has sharp diffraction spots with hexagonal structures (Fig. 2b). The interplanar spacings from these spots are calculated as *d*_1_ = 2.92 nm, *d*_2_ = 2.88 nm, and *d*_3_ = 2.67 nm. Compared with anoxic hafnium oxides predicted from ab initio calculations^37,42–44^ and h.c.p. metal Hf, these diffraction spots best fit the (10$\bar{4}$), (0$\bar{1}\bar{4}$), and ($\bar{1}\bar{1}$0) planes of h-Hf_6_O with a ($\bar{4}$4$\bar{1}$) zone axis^44^ (details are given in Supplementary Note 1). The lattice parameters of various HfO_*x*_ phases concluded in this work, with comparison to the published values in the literature, are listed in Supplementary Table 1. Similarly, adjacent region 2 is found to be m-HfO_2_ because its diffraction pattern in Fig. 2c perfectly fits the (11$\bar{1}$), (1$\bar{1}$1), and (200) planes of m-HfO_2_ with a (0$\bar{2}\bar{2}$) zone axis. Since h-Hf_6_O is a highly conductive phase, region 1 is identified as one complete CF, which supports the current flow during the SET process. The CF shows an approximately conical shape, and its terminal at the BE side is larger than that of the TE. Therefore, we infer that CFs grow from the cathode towards the anode, which is consistent with previous reports on VCM RS devices^16,30,45^. In another sample operated under 1 mA *I*_CC_, as shown in the HRTEM image of Fig. 2d, a more robust h-Hf_6_O CF (region 3) than that in Fig. 2a is found, which supports the higher ON-state current of the device. In particular, this h-Hf_6_O CF exhibits a perfect atomic arrangement and hexagonal crystal structure, as confirmed by a close-up view in the inset. According to the FFT diffraction patterns (Fig. 2e–g), the complete h-Hf_6_O CF is enclosed by the surrounding m-HfO_2_ shell (region 4). Coincidentally, crystallization of the surrounding oxide after the formation of complete CFs has also been observed in cation-based memristors^31,46^. Therefore, we propose that the CFs of HfO_2_-based RS memristors are accompanied by a nonconductive crystallization region, which has been generally ignored in previous studies on the mechanism of RS memristors.

**Fig. 3:**
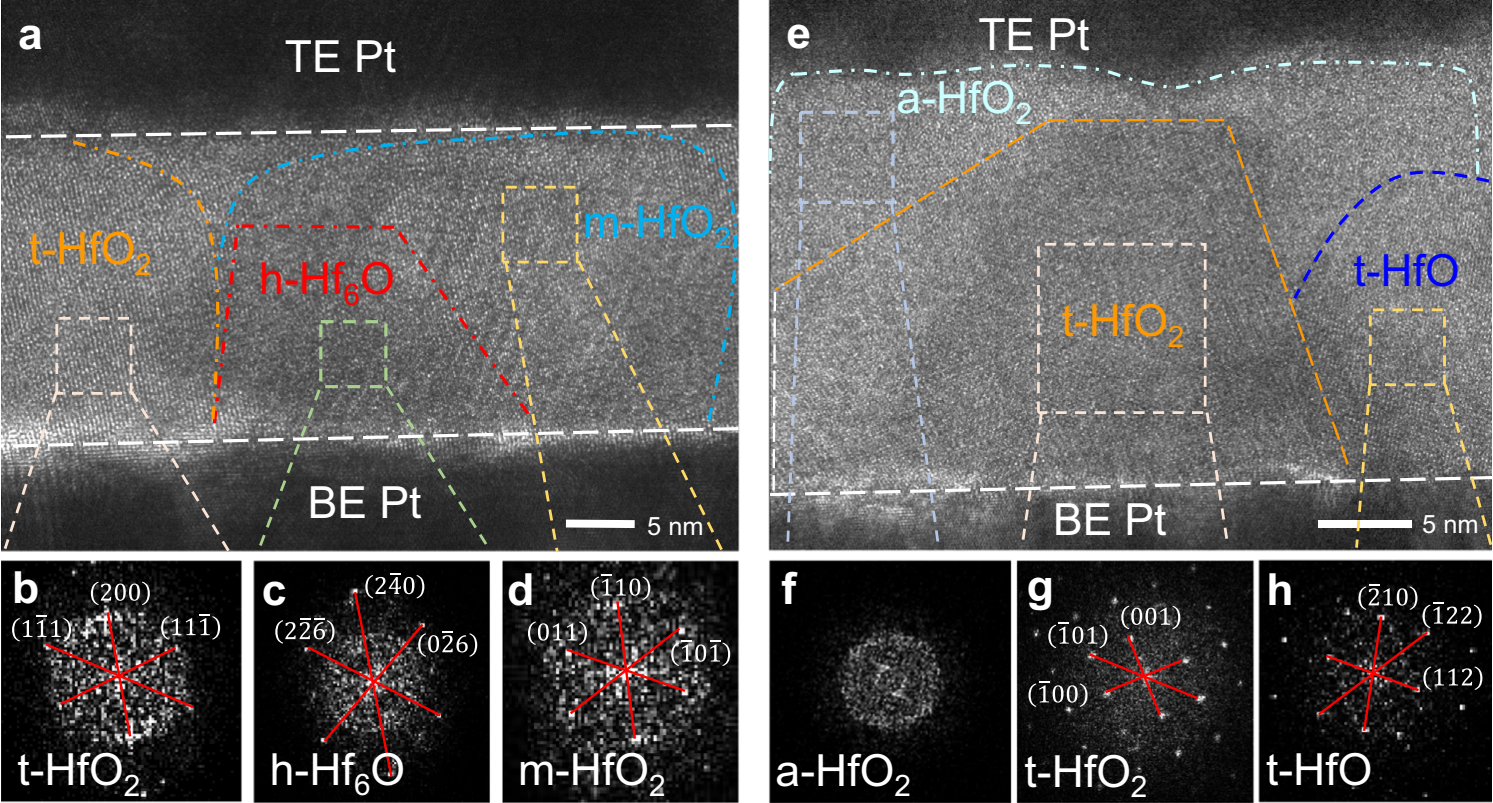
Emergence of t-HfO_2_ around the ruptured h-Hf_6_O CFs, which suffered a RESET process. **a** HRTEM image of the remaining ruptured h-Hf_6_O CF (in red) in the HRS Pt/HfO_2_/Pt RS memristor with both sides enclosed by t-HfO_2_ (in orange) and m-HfO_2_ (in blue), as confirmed by their typical FFT diffraction patterns in **b**–**d**. **e** HRTEM image of another ruptured CF region, where only t-HfO_2_ (in orange), a-HfO_2_ (in light blue), and t-HfO (in dark blue) are observed, while the CF is completely ruptured, as confirmed by their FFT diffraction patterns in **f**–**h**.

Interestingly, different crystal structures can be found around the h-Hf_6_O CFs in the Pt/HfO_2_/Pt RS memristors when the CFs were ruptured under the RESET process. Figure 3a shows an HRTEM image of an incomplete h-Hf_6_O CF region (in red) surrounded by two different crystal classes outlined by the blue and orange arcs. The FFT diffraction patterns (Fig. 3b,d) verify that the orange region is t-HfO_2_ and that the blue region is an m-HfO_2_-dominated region. As shown in Fig. 3e–h, the HRTEM and FFT diffraction patterns of one well ruptured CF region from another sample further confirm the formation of t-HfO_2_ (in orange) within the amorphous HfO_2_ RS layer (in light blue). According to Fig. 3g, the interplanar spacings are calculated as *d*_1_ = 4.93 nm, *d*_2_ = 5.15 nm, and *d*_3_ = 3.59 nm. The values of the angles between the crystal faces and the interplanar spacings are exactly the same as those of the ($\bar{1}$00), (001), and ($\bar{1}$01) planes of t-HfO_2_. In line with the typical morphology of CF growing from the BE to the TE, the t-HfO_2_ region presents a similar conical shape. It has been reported that t-HfO_2_ is a high-temperature stable phase of HfO_2_, which emerges upon heating at higher temperatures than m-HfO_2_^47,48^. These results coincide with the fact that a higher current flows through the memristor under the RESET operation and generates much more Joule heat, which is beneficial for CF rupture, than during the SET operation. The mechanism of this Joule heat-induced phase transition of HfO_x_ will be further investigated by ab initio calculations. It is interesting that t-HfO, predicted in ref.^49^ as a relatively higher-conductive phase compared to the O-rich phase of HfO_x_, emerges (in dark blue) around the ruptured CF region, as confirmed by the FFT diffraction pattern in Fig. 3h. The interplanar spacings are calculated as *d*_1_ = 2.94 nm, *d*_2_ = 3.45 nm, and *d*_3_ = 2.55 nm, best fitting the ($\bar{2}$10), (112), and ($\bar{1}$22) planes of t-HfO with the (24$\bar{3}$) zone axis^49^. Based on the above analysis, we infer that the true CF system of HfO_2_-based RS memristors includes the metallic CF and its low-conductivity shell consisting of m-HfO_2_ or t-HfO_2_ depending on the effect of Joule heat generated by current flowing through the CF.

Even though the Pt/HfO_2_/Pt memristor provides a pure system for CF study, using Pt as both the TE and the BE is not an ideal choice for industrial production. Therefore, HfO_2_-based memristors with application-oriented TEs (TiN, Ta, Hf, and Ti) were further studied to validate the scenario of the quasi-core-shell CF structure. Based on a comprehensive HRTEM study, HfO_2_-based memristors with various TEs have been demonstrated to yield a core-shell CF structure similar to that of Pt/HfO_2_/Pt devices. However, there are some subtle differences in the components of the crystalline shells. Supplementary Fig. 4a is the HRTEM image of a Pt/TiN/HfO_2_/Pt memristor (TiN-memristor) with a complete h-Hf_6_O CF region (in red) surrounded by two different crystal classes outlined by the blue and dark blue arcs. The FFT diffraction patterns in Supplementary Fig. 4b and d verify that the blue region is m-HfO_2_-dominated while the dark blue region is t-HfO-dominated. On the other hand, the shell compositions of the CF system in the Pt/Ti/HfO_2_/Pt memristor device (Ti-memristor) are m-HfO_2_ or t-HfO_2_, as shown in Supplementary Fig. 5. The shell compositions in the Pt/Hf/HfO_2_/Pt memristor (Hf-memristor) are revealed to be t-HfO_2_ and t-HfO, as shown in Supplementary Fig. 6. Finally, the Pt/Ta/HfO_2_/Pt memristor (Ta-memristor) exhibits similar CF system structure as the Hf-memristor, as shown in Supplementary Fig. 7b and d. Therefore, the quasi-core–shell structure of the CF system has universal significance for HfO_2_-based memristors.

The subtle differences in the abovementioned specific compositions of the CF system in various HfO_2_-based memristors can be attributed to the oxygen reservation capability of various TEs, as revealed by the Gibbs free energy change Δ*G* in the standard reaction of electrode oxidation (or by the bond dissociation energy of potential oxides of electrode). Relevant parameters of the Gibbs free energy and bond dissociation energy are listed in Supplementary Tables 2 and 3. A high Δ*G* value or bond dissociation energy indicates the high oxygen reservation capability of a certain metal. The formation of t-HfO_x_ has been proven more easier than that of m-HfO_x_ (x ≈ 2) in an oxygen-deficient environment^35,50^. The high oxygen reservation capabilities of Ti, Hf, and Ta electrodes result in high concentrations of oxygen vacancies in Ti-, Hf-, and Ta-memristors^15,51,52^. Therefore, the tetragonal phase preferably emerges in the Ti-, Hf-, and Ta-memristors, as demonstrated by extensive HRTEM analysis. In addition, the experimental results in this work possess an interesting consistency in that a higher oxygen reservation capability of the electrode could more likely render the emergence of a t-HfO_2_ shell, as well as a higher probability of finding *V*_Forming_ > *V*_SET_. In particular, the HRTEM images of HfO_2_-based memristors in this work provide the first experimental proof of the existence of t-HfO, which is expected as an infancy towards high-conductivity CF and deserve further explorations.

### Ab initio calculations of the HfO_x_-based CF system

**Fig. 4:**
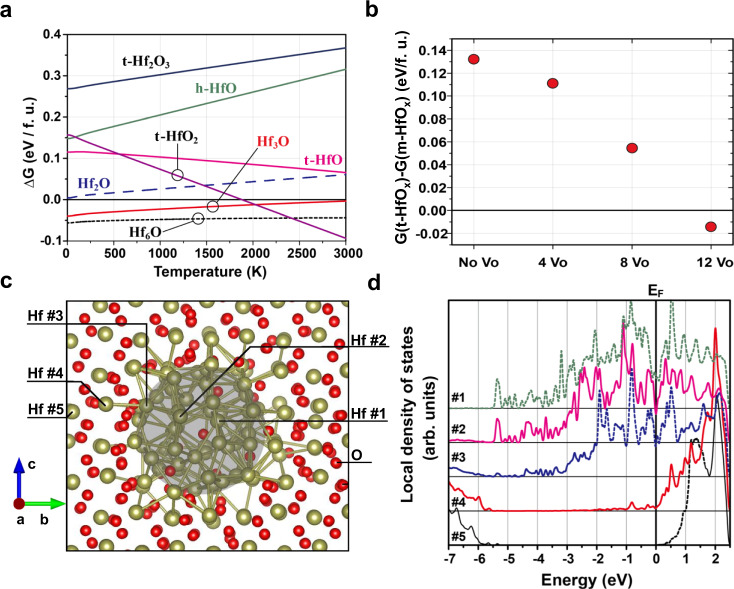
Ab initio calculations of the HfO_x_ system. **a** Relative free energy per HfO_x_ formula unit of t-HfO_2_, t-HfO, t-Hf_2_O_3_, h-HfO, h-Hf_2_O, h-Hf_3_O, and h-Hf_6_O against the decomposed h.c.p Hf and m-HfO_2_. **b** The Gibbs free energy of t-HfO_x_ with reference to m-HfO_x_ at 300 K, evaluated at several ${{{{{{\rm{V}}}}}}}_{{{{{{\rm{O}}}}}}}$ concentrations by introducing various amounts of ${{{{{{\rm{V}}}}}}}_{{{{{{\rm{O}}}}}}}$ into a Hf_32_O_64_ supercell. **c** Atomistic structure of our Hf_6_O-in-m-HfO_2_ CF model, where the Hf_6_O metal core is shaded. **d** The local density of states on several marked Hf atoms in this model with the Fermi level aligned to zero.

To obtain an elaborate view of the CF system of HfO_2_-based memristors, especially regarding the compositional evolution and structural transitions during the RS process, ab initio calculations were performed to provide evidence in terms of density functional theory and thermodynamics. Previous calculations have shown that the oxygen vacancies in HfO_2_ tend to align in an orderly manner, and this arrangement is energetically favorable for oxygen-vacancy chains to merge, thus leading to the segregation of metal Hf phases^43^. Moreover, several suboxide phases of HfO_x_ have been predicted in the literature^37,42,44,53^, such as *P*$\bar{4}$*m*2 Hf_2_O_3_ (t-Hf_2_O_3_), *P*$\bar{6}$2*m* HfO (h-HfO), and *P*$\bar{3}$1*m* Hf_2_O. Given that h.c.p. metal Hf and m-HfO_2_ are the two extreme phases for phase separation, their relative free energies (∆*G*) were taken as zero. We investigated ∆*G* of various HfO_x_ structures with temperatures ranging from 0 to 3000 K, compared with the mixture of h.c.p. Hf and m-HfO_2_ into which they may decompose. A negative relative free energy indicates a thermodynamically stable state. The vibration entropy was calculated according to statistical physics using harmonic approximation (see Supplementary Note 2 for details). As shown in Fig. 4a, the relative stability of t-HfO_2_ demonstrates the most remarkable change with temperature. In particular, this compound becomes stable against m-HfO_2_ above ~1880 K. Although this predicted phase transition temperature is slightly lower than the experimental value (~1991 K) for the 90% conversion from the monoclinic phase to the tetragonal phase^54^, the difference is within an acceptable range considering the approximations involved in our calculations. Moreover, it is clear that t-HfO_2_ gradually stabilizes against m-HfO_2_ when the sample is heated. The suboxides t-HfO, t-Hf_2_O_3_, and h-HfO are unstable at zero temperature, and the former tends to stabilize, while the latter two become even more unstable at high temperatures. The remaining three phases, Hf_6_O, Hf_3_O, and Hf_2_O, are all derivatives of h.c.p. metal Hf, with certain amounts of oxygen interstitials^44^. We first observe that Hf_2_O is unstable over the whole temperature range, while Hf_3_O is stable against decomposition into Hf and HfO_2_ at low temperatures, but its thermodynamic stability is weakened at high temperatures. The exceptional case is Hf_6_O, whose thermodynamic stability is strong over the full temperature range. Even though this compound may decompose into metal Hf and t-HfO_2_ at very high temperatures, this does not occur at less than ~2500 K.

The t-HfO_2_ phase becomes much more stable at high temperature, mainly because it is a phase with high symmetry. Thus, the entropy of t-HfO_2_ increases more rapidly with increasing temperature due to symmetry loss than the entropy of m-HfO_2_. In addition, our calculation also shows that t-HfO_x_ has a higher tolerance to oxygen vacancies (${{{{{{\rm{V}}}}}}}_{{{{{{\rm{O}}}}}}}$) than m-HfO_x_ (here, x ≈ 2). Figure 4b exhibits the Gibbs free energy differences at 300 K, with various amounts of ${{{{{{\rm{V}}}}}}}_{{{{{{\rm{O}}}}}}}$ introduced per Hf_32_O_64_ supercell. Before the stoichiometry reaches HfO_1.625_ (Hf_32_O_52_), t-HfO_x_ already becomes energetically more favorable than m-HfO_x_. In other words, the existence of a high concentration of ${{{{{{\rm{V}}}}}}}_{{{{{{\rm{O}}}}}}}$ can promote the emergence or stabilization of the tetragonal phase. This qualitative calculation coincides with our experimental observation that t-HfO_2_ is more easily observed around incomplete CFs, which has been ruptured under previous RESET operations. Oxygen anions move from the surrounding environment to the metallic CF core during the RESET process, leaving more ${{{{{{\rm{V}}}}}}}_{{{{{{\rm{O}}}}}}}^{\cdot \cdot }$ in the nearby dielectric, which can, together with the current-induced Joule heat effect, convert m-HfO_2_ to t-HfO_2_.

In addition, t-HfO_2_ possesses a much lower surface energy than m-HfO_2_^55^. We also carried out a comprehensive comparison between the surface energies from 16 kinds of m-HfO_2_ and t-HfO_2_ surface configurations (see Supplementary Note 3). For typical-shaped grains, t-HfO_2_ becomes more energetically favorable than m-HfO_2_ when each grain contains fewer than ~4000 atoms, or has an average dimension of ~3.5 nm, even without considering the entropy effect. This implies that the conversion of m-HfO_2_ to t-HfO_2_ in actual grains is easier than expected. In particular, it has been proven that, when scaled to finite size, the transformation temperature can be lowered to nearly 700 K^56,57^. In sum, the V_O_ concentration, Joule heat, and surface energy play critical roles in the evolution of the shell structure around the Hf_6_O core of the CF system. Although we cannot fully consider the nanoscale effect, the bulk calculations together with surface energy considerations still provide a useful guide towards CF formation in hafnia.

To investigate whether Hf_6_O can serve as the metallic core when surrounded by crystalline HfO_2_, we chose a model of h.c.p. Hf_6_O encapsulated by m-HfO_2_ as our core–shell CF system prototype, where the *c*-axis of Hf_6_O is aligned with the *a*-axis of m-HfO_2_. After structural relaxation using density functional theory, the optimized model structure is obtained and shown in Fig. 4c. To examine whether filamentary conduction exists in this model, we plotted the local density of states (LDOS) on a series of Hf atoms (marked as #1−#5 in Fig. 4c) in Fig. 4d. It is well known that the conduction band of HfO_2_ mainly consists of states from the metal Hf^58^. The LDOS decomposition clearly indicates that strong band conduction exists inside the cylindrical CF, as emphasized by the shaded region in Fig. 4c, and extends slightly to the surrounding region up to Hf #4. In the bulk m-HfO_2_ region, however, the system becomes insulating, as revealed by the LDOS of Hf #5. This supports that Hf_6_O inside crystalline HfO_2_ can indeed account for local filamentary conduction.

### Core-shell CF system in the HfO_2_-based memristor

**Fig. 5:**
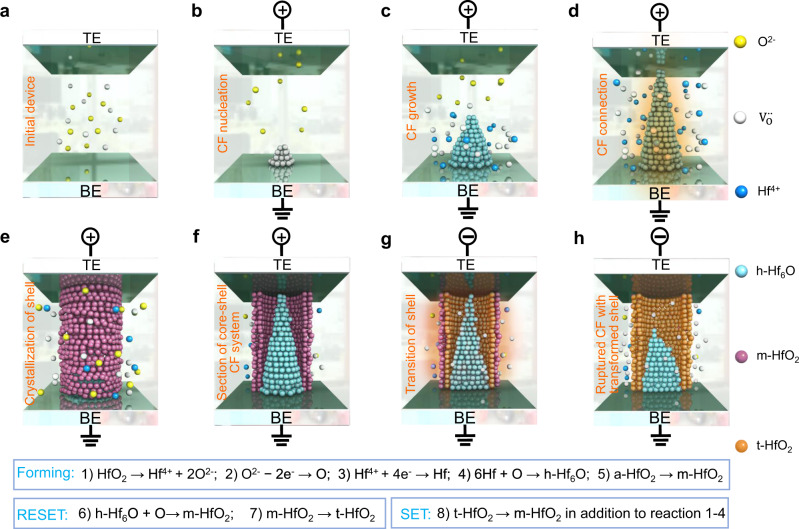
Schematic illustration of the CF system evolution in Pt/HfO_2_/Pt memristor. **a** Initial state of the device. **b** Under a positive electric field, dissociated O^2−^ ions from HfO_2_ are generated near the BE and move from the BE to the TE, resulting in localized oxygen depletion. **c** Growth of Hf-rich or O-vacancy CFs in the RS layer. **d** The as-grown CF bridge and the annealing effect of Joule heat generated by the high current. **e** The as-formed quasi-core-shell CF system consists of conductive h-Hf_6_O and the surrounding nonconductive monoclinic HfO_2_ (m-HfO_2_) shell. **f** Longitudinal-section view of the core-shell CF system. **g** Transition of the surrounding m-HfO_2_ shell into t-HfO_2_ driven by the high current through the Hf_6_O CF in the RESET process. **h** Ruptured h-Hf_6_O CF and its t-HfO_2_ shell.

Based on the above analysis, the switching mechanism and dynamic evolution of the oxygen-deficient CF system in HfO_2_-based RS memristors can be effectively updated, as schematically illustrated in Fig. 5. In the initial device, the intrinsic ${{{{{{\rm{V}}}}}}}_{{{{{{\rm{O}}}}}}}^{\cdot \cdot }$ in nonconductive a-HfO_2_ promote the formation of CFs (Fig. 5a). During the Forming process, O^2−^ ions dissociate from HfO_2_ and move from the cathode towards the anode under an external electric field, giving rise to the nucleation and growth of Hf-rich or O-vacancy CFs in the a-HfO_2_ layer (Fig. 5b, c) from the cathode to the anode. When the thermally stable anoxic h-Hf_6_O CF bridges the TE and BE (Fig. 5d), a high current flows through the CF accompanied by a device switching event from the HRS to the LRS. Under the annealing effect of current-induced Joule heat, the initial a-HfO_2_ surrounding the h-Hf_6_O CF would crystallize into m-HfO_2_ (Fig. 5e). The formation of an oxygen-rich crystalline shell is attributed to a combined effect of temperature and lateral motion of ${{{{{{\rm{V}}}}}}}_{{{{{{\rm{O}}}}}}}^{\cdot \cdot }$, which is dominated by two opposite forces: inwards thermal diffusion driven by a temperature gradient and outwards Fickian diffusion driven by a concentration gradient^59–61^ (see Supplementary Fig. 8). This finally produces a quasi-core-shell CF system as observed by HRTEM in the Pt/HfO_2_/Pt memristor. Figure 5f gives a longitudinal-section view of the quasi-core-shell structure. Reactions during core–shell CF system formation in the Forming process are dominated by the dissociation of HfO_2_ into Hf^4+^ and O^2**−**^, oxidization of O^2**−**^, reduction of Hf^4+^, combination of Hf and O into h-Hf_6_O, and crystallization of a-HfO_2_ into m-HfO_2_, as summarized by Reactions 1–5 in Fig. 5.

Despite the subtle differences in various shell structures, the shell of the CF system in HfO_2_-based RS memristors with various TEs serves as a robust shell barrier to prohibit oxygen migration towards CF owing to the fact that it is harder to create oxygen vacancies in highly crystallized oxygen-rich HfO_x_ than in amorphous HfO_2_ (supported by the oxygen vacancy formation energy calculation as shown in Supplementary Note 4). Hence, the existence of such a crystalline HfO_x_ shell helps to prohibit the CF oxidation and therefore contribute to the enhanced retention performance. Although the CF size is strongly correlated with the retention property, the crystalline shell of the CF also benefits the retention performance of the oxide memristors. This observation well explains the poor retention property of various reported low-I_CC_ memristors^62,63^, where the CF lacks a robust shell barrier to prohibit CF oxidation. In general, the quasi-core–shell CF system with excellent retention performance is expected to be one of the critical factors for RS memristor devices with extreme scalability down to sub-5 nm^64^.

Regarding the RESET process, the oxygen ions formed by reduction reaction at the TE/RS interface are driven back by an electric field and react with the h-Hf_6_O CF to generate nonconductive HfO_2_. Moreover, part of the CF fuses with the assistance of Joule heating (so-called TCM-based RESET process). Both Joule heating and electric field play important roles in the RESET process, when the device switches from the LRS to the HRS. As evidenced by the electrical measurement, a much higher current is needed to switch the device from the LRS back to HRS. Therefore, the Joule heat effect on the h-Hf_6_O CF is much more significant in the RESET process than in the Forming process. Accordingly, the surrounding m-HfO_2_ shell transforms into t-HfO_2_ (Fig. 5g), which is a high-temperature stable phase of HfO_2_ with a high formation energy^65,66^, and the h-Hf_6_O CF starts to rupture at its thinnest part near the TE side, leaving a conical residue (Fig. 5h). Additionally, the rupture process of the h-Hf_6_O CF draws back oxygen from its surroundings, contributing to the existence of abundant ${{{{{{\rm{V}}}}}}}_{{{{{{\rm{O}}}}}}}$ in the crystalline t-HfO_2_ shell. The loss of oxygen in the t-HfO_2_ shell region further promotes its stability (as t-HfO_x_) and prohibits its transition to room temperature stable m-HfO_2_ after the RESET process, as revealed by the relative free energy in the ab initio calculations (Fig. 4b). Reactions during the RESET process are summarized as Reactions 6 and 7 in Fig. 5. The reactions in the following SET operation are similar to those in the Forming process, while the t-HfO_2_ shell outside the h-Hf_6_O core may transform back to m-HfO_2_ owing to the decrease in ${{{{{{\rm{V}}}}}}}_{{{{{{\rm{O}}}}}}}$ concentration (Reaction 8). There is a tradeoff for the *V*_SET_ of RS memristors: local electric field enhancement of the CF residue helps decrease *V*_SET_^67^, while the formation of CF in the highly crystalline package layer needs a higher *V*_SET_ than in the amorphous structure^68^. Considering the probability event of *V*_SET_ > *V*_Forming_ in HfO_2_-based RS memristors with various TEs (Supplementary Fig. 2), the crystalline environment, as well as the weak oxygen reservation capability of the electrode, explain the higher *V*_SET_ of the Pt/HfO_2_/Pt RS memristor than its *V*_Forming_.

In summary, we have studied the dynamic changes in the physical properties of the CF system in oxide memristors, including its composition, structure, and especially the evolution of the CF surroundings based on Pt/HfO_2_/Pt devices with a-HfO_2_ as the RS layer. The Pt/HfO_2_/Pt devices exhibit considerable RS performance, including a large switching window (HRS/LRS) above 10^6^, good retention, and a short OFF/ON switching time within 120/20 ns. By analyzing the atomic structure of the CFs and their surroundings using HRTEM, we conclude that the CF system in HfO_2_-based memristors is a quasi-core–shell structure: the center of the CF system consists of metallic h-Hf_6_O surrounded by nonconductive m-HfO_2_, t-HfO_2_, or poorly conductive t-HfO. The core-shell CF system has universal significance for HfO_2_-based memristors, even though the specific components of the crystalline shell vary with the oxygen reservation capability of various TEs. It is demonstrated that the concentration of oxygen vacancies, Joule heat, and surface energy play critical roles in the evolution/transition of the shell structure around the h-Hf_6_O core of the CF system. The quasi-core–shell CF system with an intrinsic barrier, which prohibits CF oxidation and ensures good retention performance, is believed to be one of the critical factors for the extreme scalability of the RS memristor devices down to sub-5 nm. This study renders a further understanding of the nature of the CF system, deepens the VCM and TCM mechanism of HfO_2_-based memristors, and provides potential inspirations to improve oxide-based RS memristors for memory applications.

## Methods

### Sample preparation

The detailed fabrication processes of the crossbar Pt/HfO_2_/Pt and HfO_2_-based RS memristors with various electrodes (TiN, Ta, Hf, and Ti) are illustrated in Supplementary Fig. 1. The flake samples of the Pt/HfO_2_/Pt memristor for TEM characterization were prepared by the focused ion beam (FIB) technique (FEI Helios Nanolab 450s, UK). After the deposition of carbon and platinum protecting layers, the target regions of the samples were etched to a thickness of 50 nm for electron transmission.

### Characterizations

The electrical characteristics, including *I*–*V* curves, retention, and endurance of the Pt/HfO_2_/Pt memristors, were measured at room temperature using an Agilent B1500A Semiconductor Device Analyzer under DC sweep mode. The speed characteristics of the memristors were implemented under pulse mode in the atmosphere. A waveform generator/fast measurement unit module (WGFUM) in the Agilent B1500A Analyzer was used to generate the voltage pulse and measure the response current at the same time in this experiment. The temperature–resistance characteristics of the LRS and the HRS samples were measured by a Keithley 4200-SCS semiconductor characterization system with the temperature changing from 180 K to 400 K under vacuum. For all *I*–*V* sweeps and pulse mode experiments, the bias was always applied to the TE, and the BE was grounded.

### SEM and HRTEM experiments

An SEM image of the Pt/HfO_2_/Pt memristor was obtained using a field-emission scanning electron microscopy (ZEISS SUPRA 55 SAPPHIRE). The flakelet samples of the Pt/HfO_2_/Pt memristor for TEM characterization were prepared by the FIB etching technique (FEI Helios Nanolab 450s, UK). The target region of the sample was milled to 50 nm in thickness for electron transmission. TEM images were obtained with a field-emission gun/TEM (FEI Tecnai TF-20, UK) operated under 200 kV voltage. The FFT diffraction patterns were analyzed by TEM Imaging & Analysis (TIA, FEI) software.

### Ab initio calculation

Density functional calculations were carried out using the projector augmented-wave (PAW) method, with the Vienna Ab Initio Simulation Package (VASP 5.4.4)^69,70^. A plane wave basis set with 500 eV kinetic energy cutoff was chosen to expand the wave-functions. Generalized gradient approximation (GGA) was adopted for the exchange-correlation energy, in the simple Perdew–Burke–Ernzerhof (PBE) functional form^71^. In Gibbs free energy calculations, we chose the valence electrons as: 5s, 5p, 5d, and 6s for Hf; 2s and 2p for O, while in filament-in-dielectric supercell calculations, the 5s and 5p electrons were considered as in the core part of the Hf pseudopotential. The vibration frequencies were calculated using the density functional perturbation theory, while the vibration entropies were derived using the harmonic oscillator model. On account of the semiconductor band gap problem due to GGA, we adopted the self-energy corrected GGA-1/2 method^72,73^ in electronic structure calculations for the filament-in-dielectric supercell, which fits normal oxides like HfO_2_. The optimum cutoff radius for the O PBE self-energy potential^74^ was calculated to be 2.7 bohr in HfO_2_, through a variational method. No empirical parameter was involved in the GGA-1/2 calculation. The GGA-1/2 electronic structure for monoclinic HfO_2_ is comparable with that of the Heyd-Scuseria-Ernzerhof (HSE06) hybrid functional result (see Supplementary Fig. 12).

### Statistics and reproducibility

Experiments were reproducible.

Figure 1b, the experiments were performed five times with similar results.

Figure 2a, d, the experiments were performed six times with similar results.

Figure 3a, e, the experiments were performed six times with similar results.

Supplementary Figure 4a, the experiments were performed once.

Supplementary Figure 5a, the experiments were performed twice with similar results.

Supplementary Figure 6a, the experiments were performed once.

Supplementary Figure 7a, the experiments were performed once.

### Reporting summary

Further information on research design is available in the Nature Research Reporting Summary linked to this article.

## Supplementary information

Supplementary Information

Reporting Summary

## Data Availability

The data that support the plots within this paper and other findings of this study are available from the corresponding authors upon reasonable request.

## Code availability

All code used in this study is available from the corresponding authors upon reasonable request.
