# Supplementary material for: Evolution of the conductive filament system in HfO2-based memristors observed by direct atomic-scale imaging
Source: Nat Commun. 2021 Dec 13;12:7232. doi: 10.1038/s41467-021-27575-z (PMC8668918; doi:10.1038/s41467-021-27575-z)
Supplement: Supplementary file 1 — Supplementary Information [file 41467_2021_27575_MOESM1_ESM.pdf]

Supplementary Information

Evolution of the conductive filament system in HfO_2-based memristors observed by direct atomic-scale imaging

Ying Zhang[1,2,3], Ge-Qi Mao[4], Xiaolong Zhao[2*], Yu Li[1], Meiyun Zhang[1,3], Zuheng Wu[1,3], Wei Wu[4], Huajun Sun[4], Yizhong Guo[5], Lihua Wang[5], Xumeng Zhang[1,3,6], Qi Liu[1,3,6], Hangbing Lv[1,3], Kan-Hao Xue[4*], Guangwei Xu[2], Xiangshui Miao[4], Shibing Long[2*], and Ming Liu[1,3,6*]

[1] Key Laboratory of Microelectronic Devices & Integration Technology, Institute of Microelectronics of Chinese Academy of Sciences, Beijing 100029, China.

[2] School of Microelectronics, University of Science and Technology of China, Hefei 230026, China.

[3] University of Chinese Academy of Sciences, Beijing 100049, China.

[4] School of Integrated Circuits, School of Optical and Electronic Information, Huazhong University of Science and Technology, Wuhan 430074, China.

[5] Institute of Microstructure and Property of Advanced Materials, Beijing Key Laboratory of Microstructure and Property of Advanced Materials, Beijing University of Technology, Beijing 100124, China.

[6] Frontier Institute of Chip and System, Fudan University, Shanghai 200433, China.

[*]e-mail: xlzhao77@ustc.edu.cn; xkh@hust.edu.cn; shibinglong@ustc.edu.cn; liuming@ime.ac.cn

Supplementary Figures

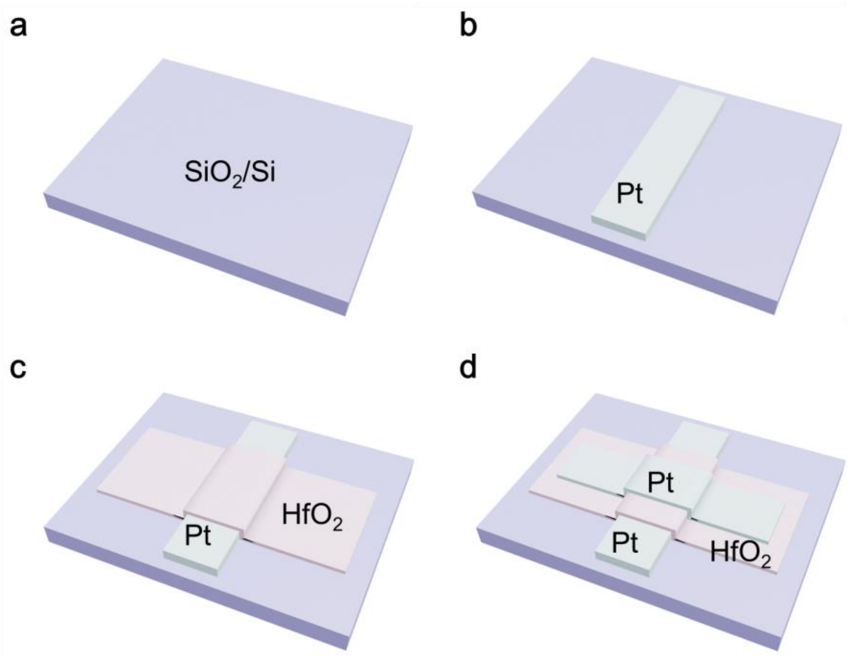

Supplementary Figure 1 | Fabrication process of the crossbar Pt/HfO_2/Pt memristors. a, Clean SiO_2/Si substrate for memristor fabrication. **b**, After the first photolithography process, 5 nm Ti adhesion layer and 40 nm Pt BE film were successively deposited onto the substrate by radio-frequency magnetron sputtering method, followed by a lift-off process to form the patterned BE lines. **c**, After the second photolithography process, 20 nm HfO_2 RS film was sputtered (Ar 20 sccm, O_2 0.2 sccm, power 60 W) at room temperature using a HfO_2 target, followed by a lift-off process. **d**, After the third photolithography process, 30 nm Pt TE film was prepared under the same conditions of TE film, followed by a lift-off process to release the memristor devices. The effective area of the crossbar device is 3×3 μm^2. HfO_2-based RS memristors with different electrodes (TiN, Ti, Hf, and Ta) were prepared by the similar method.

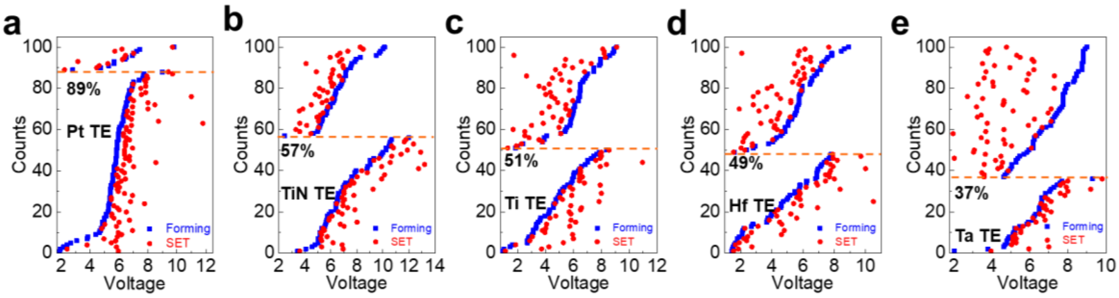

Supplementary Figure 2 | Statistical data of $V_{Forming}$ and 1st V_{SET} (after 1st RESET operation) from randomly selected 100 cells for each HfO_2-based memristors with different top electrodes (TEs), including a, Pt/HfO_2/Pt; b, Pt/TiN/HfO_2/Pt; c, Pt/Ti/HfO_2/Pt; d, Pt/Hf/HfO_2/Pt; and e, Pt/Ta/HfO_2/Pt devices. V_{SET} is higher (lower) than $V_{Forming}$ below (above) the orange dashed line in the figures. During the measurement, the stop voltage of RESET operation was fixed at −4.5 V.

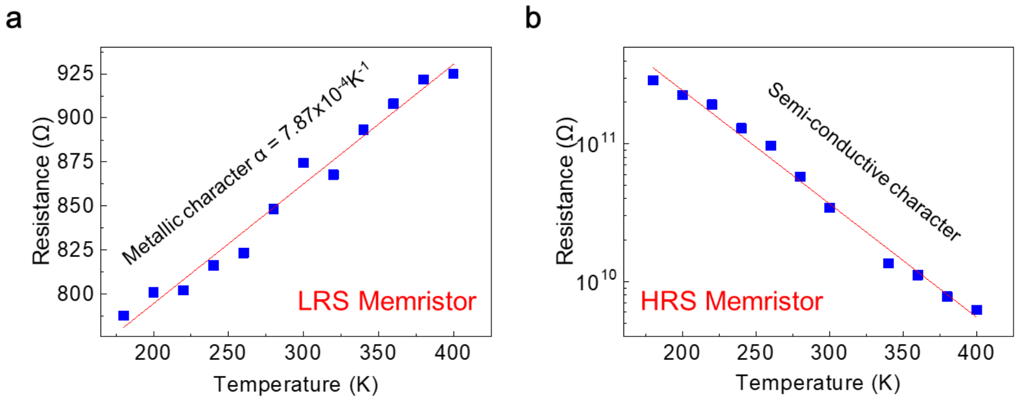

Supplementary Figure 3 | A temperature-resistance measurement (180 K to 400 K) of the LRS and HRS memristors to further confirm their conduction characteristics. **a**, Temperature dependence of the LRS of the memristor. LRS increases linearly with increment of surrounding temperature, indicating that the CFs are with metallic conduction. The resistance temperature coefficient of the LRS device is 7.87×10^{-4} K^{-1}, comparable with but lower than that of the reported value (3.9×10^{-3} K^{-1}) of pure hafnium nanowire. **b**, Temperature dependence of the HRS of the memristor. The HRS decreases with the increasing temperature, indicating a semiconductive characteristic.

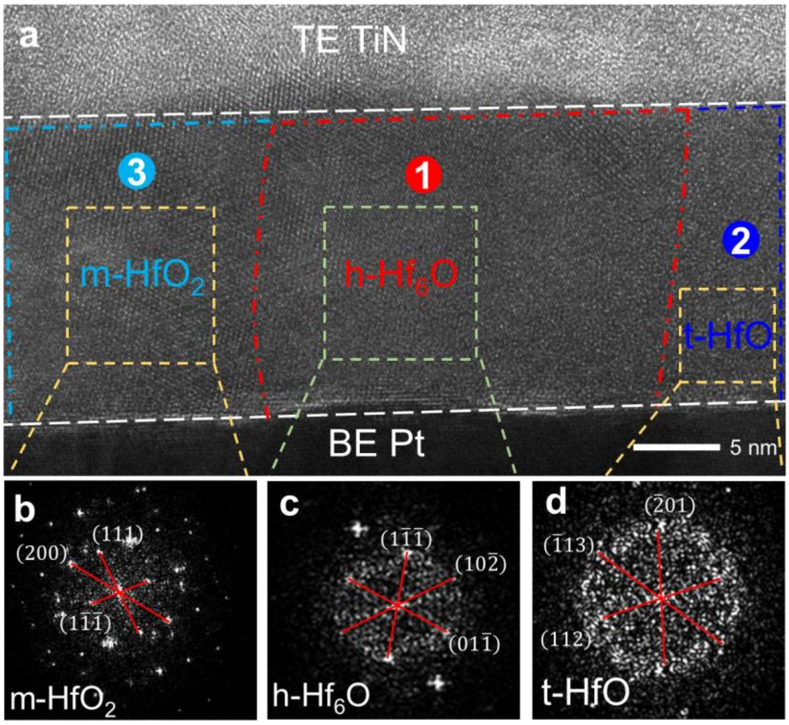

Supplementary Figure 4 | Complete CF and its crystalline shells in LRS Pt/TiN /HfO_2/Pt RS memristors. a, HRTEM of a complete CF in the LRS device operated under 0.1 mA I_{CC} with the typical polymorphous HfO_x region, namely, m-HfO_2, h-Hf_6O and t-HfO region, as confirmed by their FFT diffraction patterns in **b, c** and **d**.

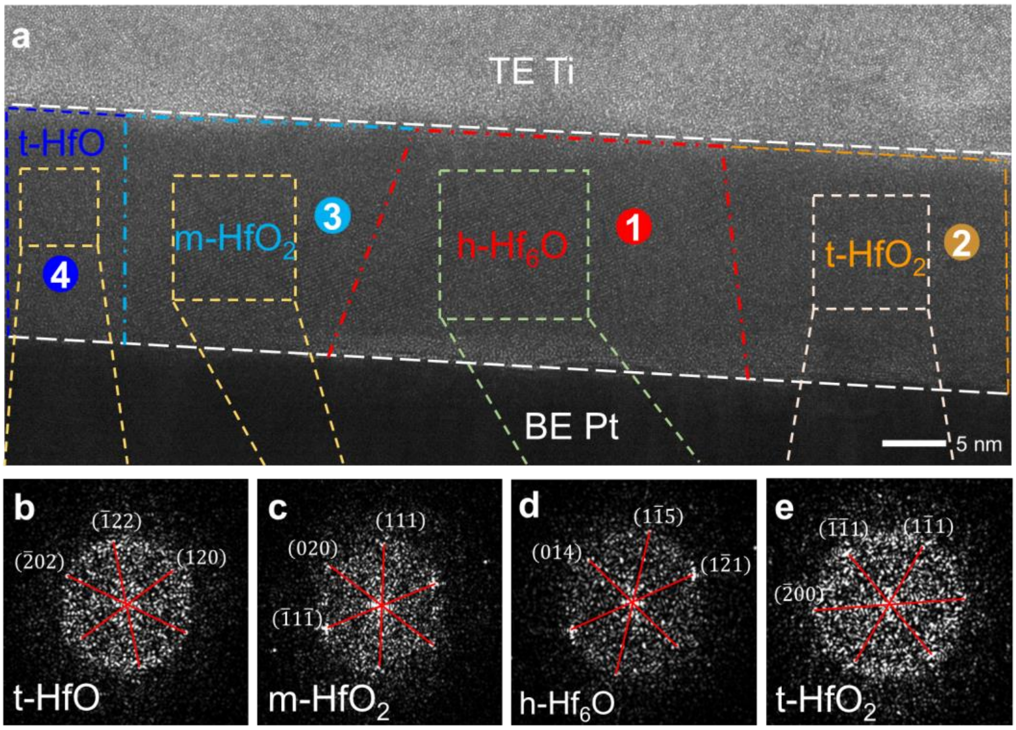

Supplementary Figure 5 | Complete CF and its crystalline shells in LRS Pt/Ti/HfO_2/Pt RS memristors. a, HRTEM of a complete CF in the LRS device operated under 0.1 mA I_{CC} with the typical polymorphous HfO_x region, namely, t-HfO, m-HfO_2, h-Hf_6O, and t-HfO_2 and region, as confirmed by their FFT diffraction patterns in **b, c**, **d** and **e**.

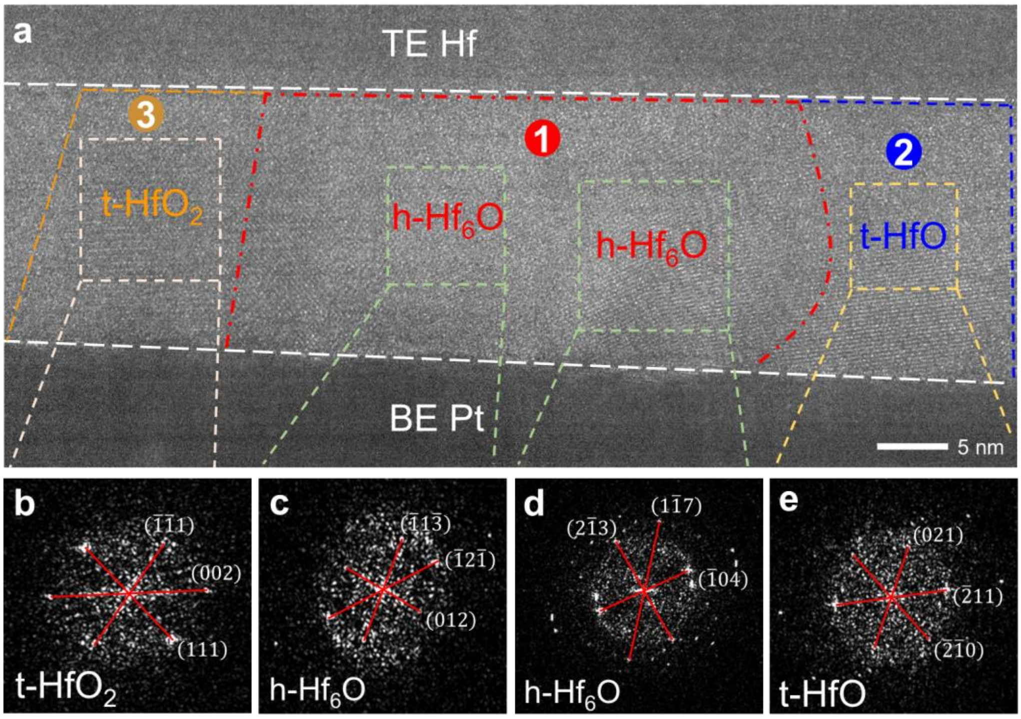

Supplementary Figure 6 | Complete CF and its crystalline shells in LRS Pt/Hf/HfO_2/Pt RS memristors. a, HRTEM of a complete CF in the LRS device operated under 0.1 mA I_{CC} with the typical polymorphous HfO_x region, namely, t-HfO_2, h-Hf_6O and t-HfO region, as confirmed by their FFT diffraction patterns in **b, c, d and e.**

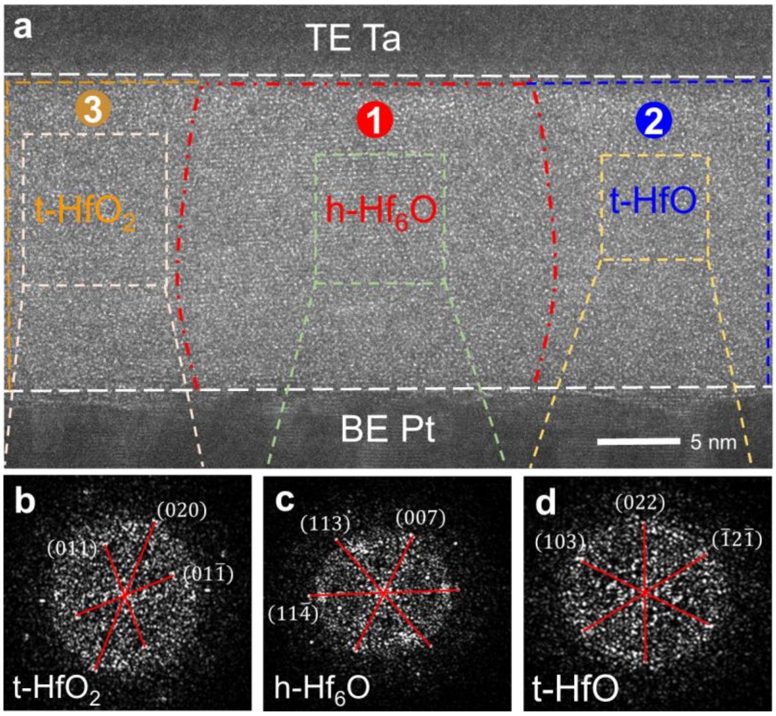

Supplementary Figure 7 | Complete CF and its crystalline shells in LRS Pt/Ta/HfO_2/Pt RS memristors. a, HRTEM of a complete CF in the LRS device operated under 0.1 mA I_{CC} with the typical polymorphous HfO_x region, namely, t-HfO_2, h-Hf_6O and t-HfO region, as confirmed by their FFT diffraction patterns in **b, c** and **d**.

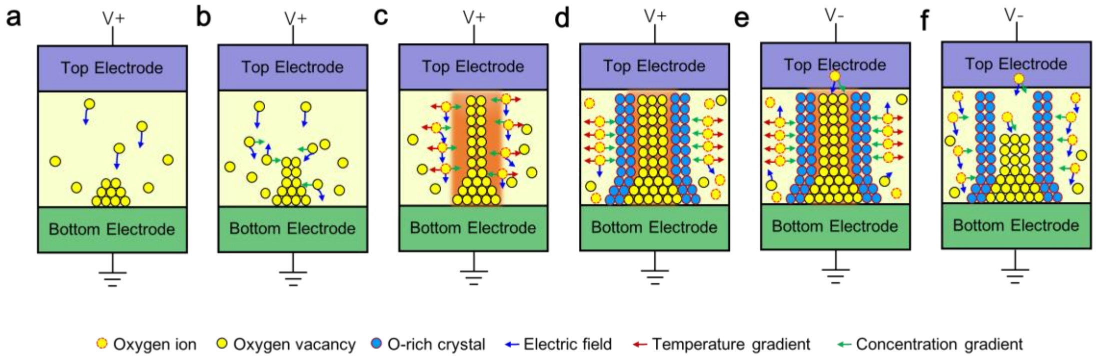

Supplementary Figure 8 | Schematic illustration of oxygen-ions motion during SET and RESET process. The electric field dominates the vertical motion, hence the other two forces driven by temperature and concentration gradient, are only analyzed in the lateral direction. a, Oxygen ions move from BE to TE under electric field. **b,** Concentration gradient formed by oxygen-vacancy clusters accumulation moves oxygen ions toward CF before it reaches the top electrode. **c,** Temperature

gradient by CF Joule heating moves oxygen ions outward CF. **d,** O-rich crystalline shell formed assisted by the lateral motion of oxygen-ions and the Joule heating effect. **e,** In regard to RESET process, electric-field moves oxygen ions from TE to BE, while the directions driven by temperature and concentration gradient remain unchanged. **f,** Temperature gradient weakens after dissolution of CF, while the effects of electric field and concentration gradient still exist.

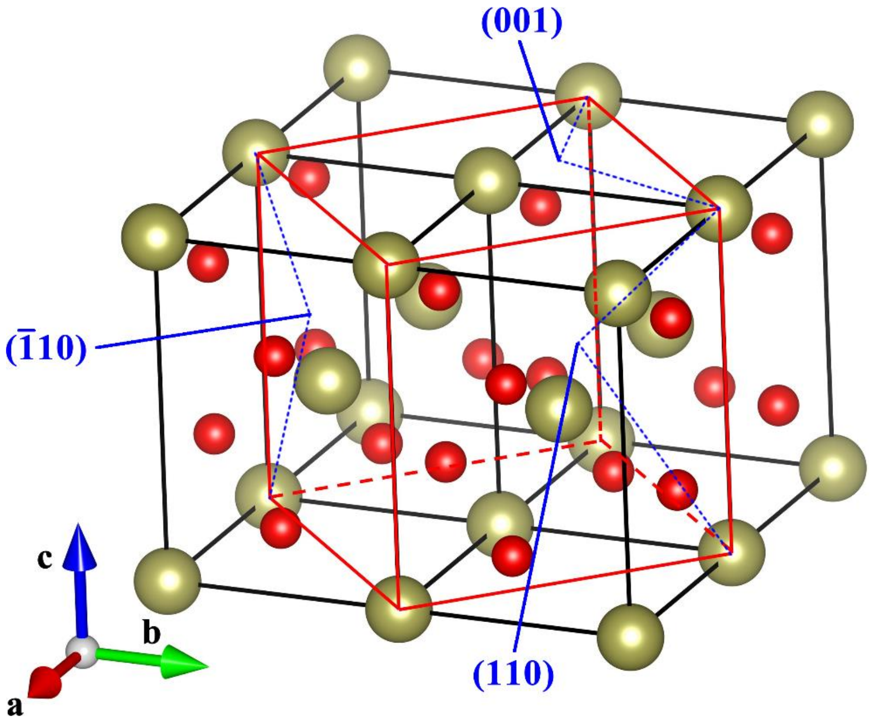

Supplementary Figure 9 | Schematic view of the selected grain in tetragonal HfO_2. The figure shows a $\sqrt{2}\times\sqrt{2}$ supercell of tetragonal HfO_2, where the grain is embedded as a tetragonal box with red line color. Various surfaces of the grain including (001), ($\bar{1}$10) and (110) are marked by blue lines.

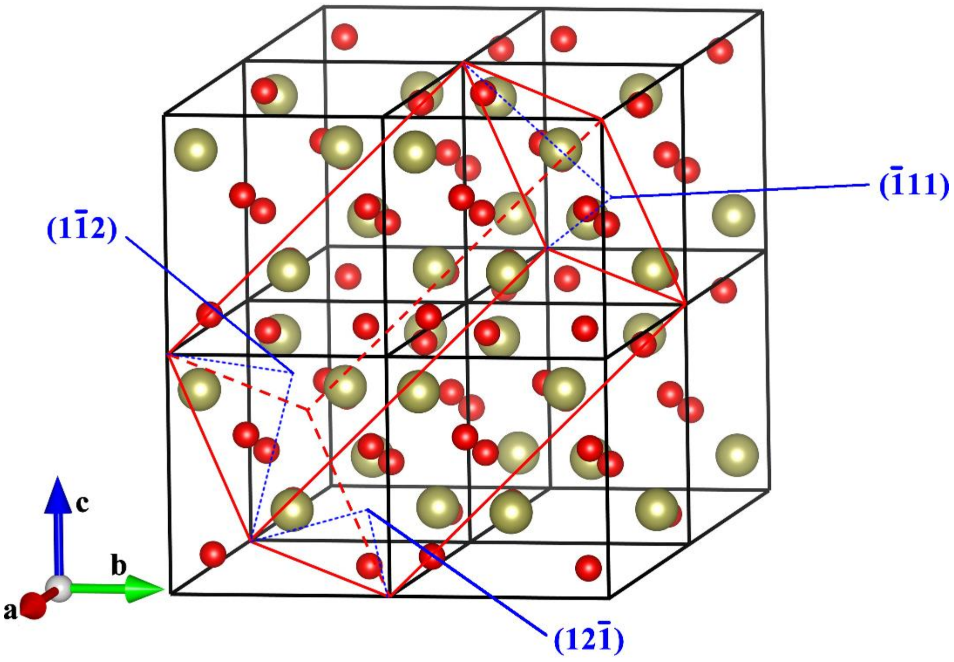

Supplementary Figure 10 | Schematic view of the selected grain in monoclinic HfO_2. The figure shows a 96-atom supercell of monoclinic HfO_2, where the grain is embedded as a box with red line color. Various surfaces of the grain including ($\bar{1}$11), (1$\bar{1}$2) and (12$\bar{1}$) are marked by blue lines.

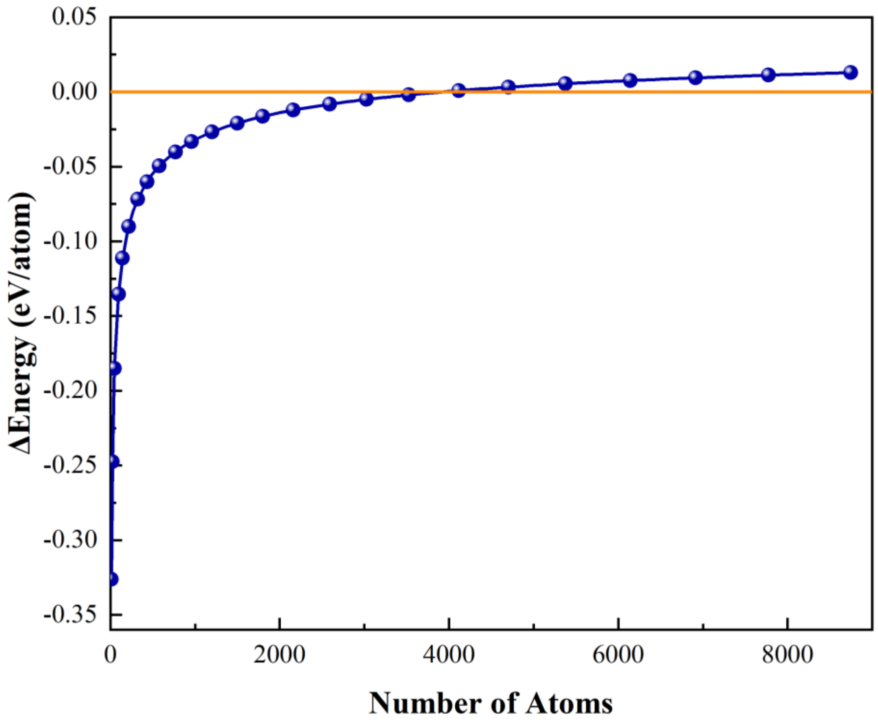

Supplementary Figure 11 | Energy comparison between hafnia grains of different phases and sizes. The relative energies of a tetragonal hafnia grain with respect to a monoclinic hafnia grain with the same number of atoms are plotted against the number of atoms.

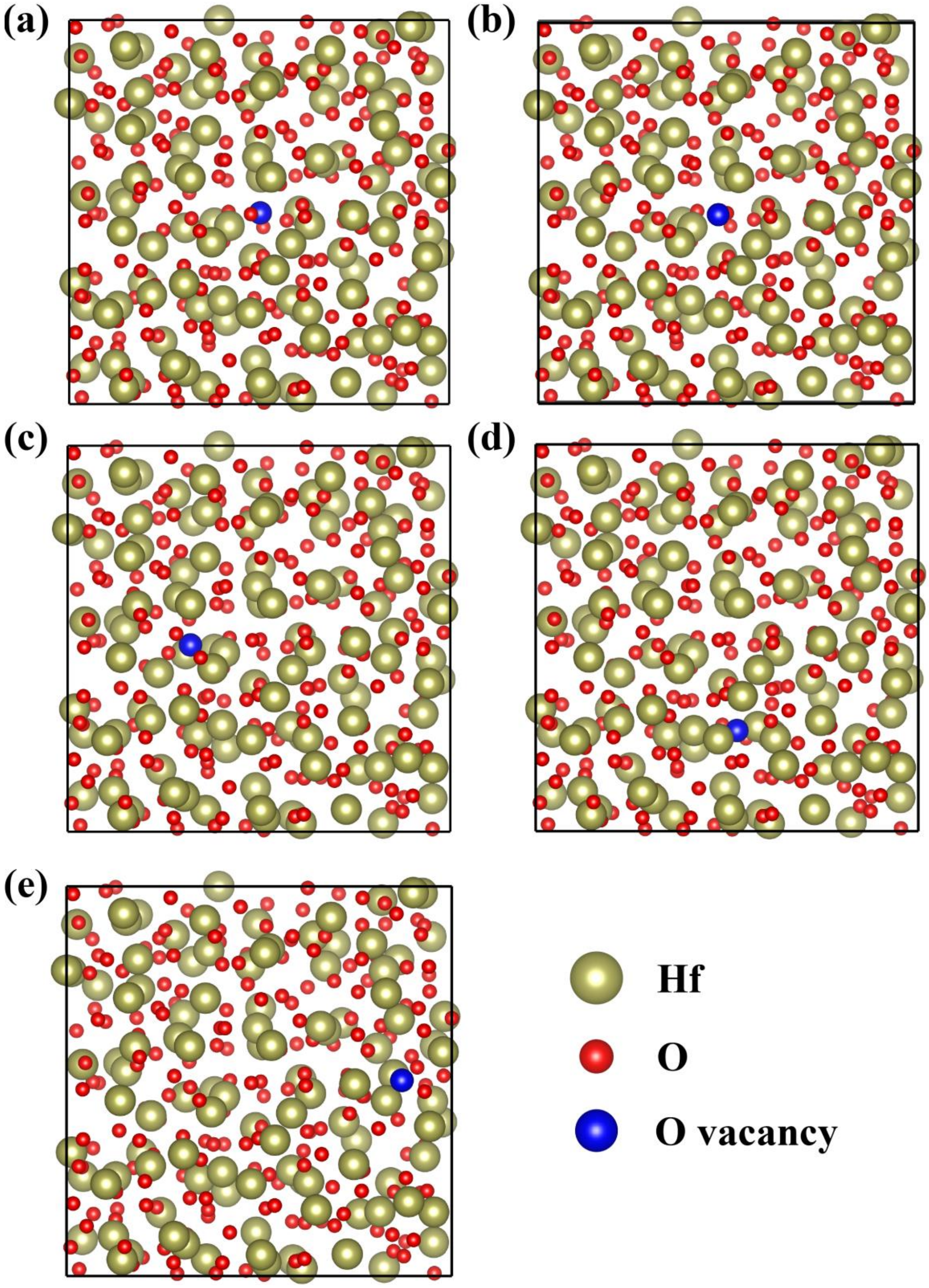

Supplementary Figure 12 | Illustration of the oxygen vacancy sites under investigation. Five distinct oxygen vacancy sites are demonstrated, derived from the same amorphous HfO_2 supercell with 324 atoms. **a**, Case 1; **b**, case 2; **c**, case 3; **d**, case 4; **e**, case 5.

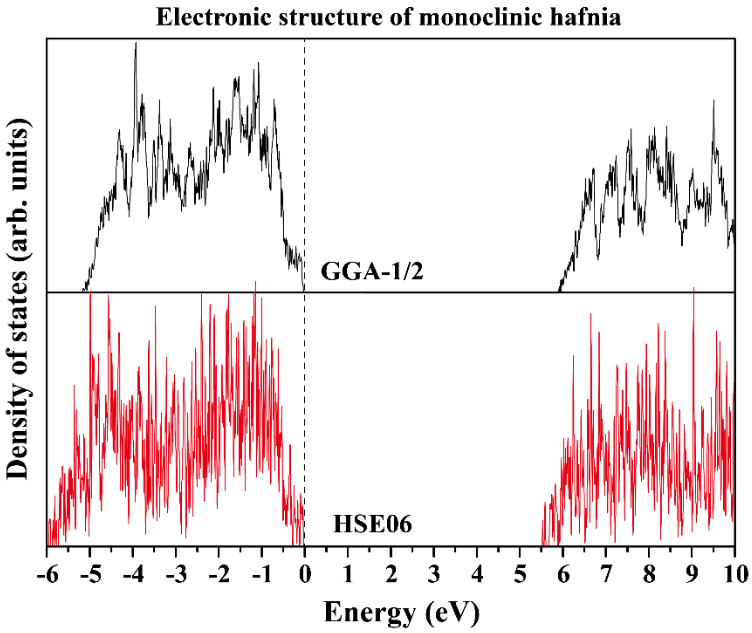

Supplementary Figure 13 | Electronic density of states for monoclinic HfO_2, calculated using GGA-1/2 and the HSE06 hybrid functional, showing similar band gaps and valence/conduction band morphology. t-HfO_2 becomes more energetically favorable than m-HfO_2 when the grain contains less than ~4000 atoms.

Supplementary Tables

Supplementary Table 1 | Lattice parameters of various Hf-O phases concluded in this work, with comparison to the published values in the literature. Length unit: Å; angle unit: degree.

Phase	This	Experimental	Calculated

	work							
m-HfO_2				300K	940K	VASP	SIESTA	CASTEP
a	5.146	5.117[1]	5.119[2]	0.512[3]	0.5110[3]	5.1322[4]	5.10[5]	5.08[6]
b	5.203	5.175	5.170	0.5172	0.5168	5.1887	5.17	5.19
c	5.328	5.291	5.298	0.529	0.5282	5.3067	5.26	5.22
β	99.65	99.22	99.18	99.5	99.14	99.78	99.12	99.77
t-HfO_2						VASP	SIESTA	CASTEP
a	5.083	5.04~5.10[7]	5.14[8]	5.15[2]		5.06[4]	5.03[5]	3.56[6]
c	5.225	5.11~5.12	5.25	5.29		5.18	5.18	5.11
t-Hf_2O_3								
a	3.135					3.135		
c	5.657					5.646		
t-HfO								
a	6.658					6.648228[9]		
c	10.388					10.36824		
h-HfO								
a	5.231					5.226[10]		
c	3.191					3.172		
h-Hf_2O								
a	5.582							
c	5.145							
h-Hf_3O								
a	5.582							
c	15.36							

	0		

h-Hf$_6$O			
a	5.553	5.559	
c	15.34<br>1	15.387[11] 14.4~<br>1<br>5.6[12]	

Hf			
a	3.196	3.1946[13] 3.1964<br>8 PDF#38-1478	3.077[14] 3.201[15]
c	5.055	5.0510 5.058	4.905 5.061

Supplementary Table 2 | List of Gibbs free energies of related substance potentially formed by oxidation of different top electrodes (The Gibbs free energies are extracted from Lange's Handbook of Chemistry, 70th Anniversary Edition, 16th 2004).

Substance	ΔG_f ((kJ mol^{-1})
Ti_3O_5	−2317.4
Ti_2O_3	−1434.2
TiO_2	−888.8
TiO	−495.0
HfO_2	−1088.2
Ta_2O_5	−1911.0
TaO_2	−209.0

* The Gibbs free energies are extracted from Lange's Handbook of Chemistry, 70th Anniversary Edition, 16th 2004.

Supplementary Table 3 | List of bond dissociation energies of possible metal-oxygen (M-O) bonds in this work.

Bond	ΔHf_{298}(kJ mol^{-1})
Pt-O	347

Ti-O	662
Hf-O	791
Ta-O	805

* The Gibbs free energies are extracted from Lange's Handbook of Chemistry, 70th Anniversary Edition, 16th 2004.

Supplementary Table 4 | Spacing calculation of Fig. 2b.

	d_1	d_2	d_3
$d_{mearsured}$/nm	2.915452	2.881844	2.666667
d h-Hf/nm	2.767817	2.767817	2.767817
Error%	5.06387	3.956744	3.793145
d m-HfO_2/nm	2.824074	3.144979	2.610000
Error%	3.134262	9.130771	2.125000
d h-Hf_6O/nm	2.964617	2.964617	2.744135
Error%	1.686369	2.872216	2.905063
d t-HfO_2/nm	2.953999	2.953999	2.608070
Error%	1.322166	2.503765	2.197375
d t-HfO/nm	2.877642	2.877642	2.558490
Error%	1.296879	0.145823	4.056628

Supplementary Table 5 | Crystal plane angle of Fig. 2b.

	h_1	k_1	l_1	h_2	k_2	l_2	Angle (°)	Angle_measured (°)	Error%
h-Hf	−1	0	0	0	1	0	120.00	114.96	4.384134
	−1	0	0	−1	1	0	60.00	58.20	3.092784
	0	1	0	−2	2	1	60.00	56.76	5.708245
m-HfO_2	1	1	1	0	0	−2	129.20	114.96	12.38979
	1	1	1	1	1	−1	69.35	58.20	19.14966
	0	0	−2	1	1	−1	59.85	56.76	5.330162
h-Hf_6O	−1	0	4	0	−1	−4	114.61	114.96	0.305237
	−1	0	4	−1	−1	0	57.31	58.20	1.538488
	0	−1	−4	−1	−1	0	57.30	56.76	0.959479
t-HfO_2	1	1	1	0	0	−2	124.49	114.96	8.293232
	1	1	1	1	1	−1	68.99	58.20	18.53591
	0	0	−2	1	1	−1	55.50	56.76	2.209126
t-HfO	1	0	−1	0	0	2	122.58	114.96	6.628392
	1	0	−1	1	0	1	65.17	58.20	11.97595
	0	0	2	1	0	1	57.41	56.76	1.145172

Supplementary Table 6 | List of calculated surface energies for various tetragonal/monoclinic HfO_2 surfaces.

Face	Surface energy (J/m^2)
Tetragonal HfO_2	
(001)	1.053
(100)	1.834
(110)	1.105
(101)	1.128
(111)	2.229
Monoclinic HfO_2	
(001)	1.354
(010)	2.056
(100)	1.949
(110)	1.763
(101)	1.819
(011)	1.737
($\bar{1}$01)	1.958
(111)	1.224
($\bar{1}$11)	1.063
(12$\bar{1}$)*	1.821
(1$\bar{1}$2)*	1.797

*These two surfaces are orthogonal to the ($\bar{1}$11) surface of monoclinic hafnia.

Supplementary Table 7 | List of oxygen vacancy formation energies for various sites in monoclinic and amorphous HfO_2.

Phase	Oxygen vacancy formation energy (eV)				
Monoclinic	III-coordination	IV-coordination			
	6.39	6.26			
Amorphous	Site (a)	Site (b)	Site (c)	Site (d)	Site (e)
	6.33	6.15	5.58	6.06	6.19

Supplementary Notes

Supplementary Note 1: Detailed description of phase determination

In order to identify the phases present in each region of the HRTEM patterns, we calculated crystal parameters (values of the angles between the crystal faces and the interplanar spacings) based on our FFT diffraction pattern, and then compared these parameters with previously published experimental data and theoretical calculation results (Supplementary Table 4 and 5). Based on the comparison, the exact phase that best fits our experimental data can be identified.

Take Region 1 in Fig. 2a as an example. The interplanar spacings from these spots in Fig. 2b are calculated as d_1 = 2.92 nm, d_2 = 2.88 nm, and d_3 = 2.67 nm, and the crystal plane angles are 114.96°, 58.20°, and 56.76°, respectively. Interplanar spacings and crystal plane angles of the possible HfO_x phases, which approximately match these experimental parameters, are listed in Supplementary Table 4 and 5, respectively. It is obvious that both the closest interplanar spacings (Supplementary Table 4) and crystal plane angles (Supplementary Table 5) of h-Hf exhibit relative larger errors (max error reaching 5.708%) than those of h-Hf_6O (max error merely 1.538%) when matching the experimental parameters. On the other hand, t-HfO_2 can be excluded because of its large error in plane angles, though it shows the smallest errors in the interplanar spacings. Based on all above analysis, h-Hf_6O is the best matching suboxide phase of Fig. 2b, considering both the interplanar spacing and crystal plane angle.

For the situation in Fig. 2f, the interplanar spacings are calculated as d_1 = 4.09 nm, d_2 = 2.95 nm, d_3 = 2.57 nm. Compared with PDF #38-1478 card of h.c.p. Hf, no appropriate planes can fit these diffraction spots and match the Vector law under the interplanar spacing error within 10%. The FFT diffraction spots can be clearly calibrated as h-Hf_6O rather than h.c.p Hf by the same analysis as in Fig. 2b. After careful comparison, the core of CF system regions discussed in the revised manuscript all best match with h-Hf_6O.

Supplementary Note 2: Vibration entropy calculation

Using the harmonic oscillator model, we write the energy eigenvalues in each degree of freedom as:

$$\varepsilon_n = \left(n + \frac{1}{2}\right) hv$$

where v is the vibration frequency calculated with the density functional perturbation theory. The zero point energy is simply as

$$ZPE = \frac{h}{2}\sum_{i=1}^{3N} v_i$$

where N is the number of atoms and the sum is thus carried out over all degrees of freedom. The zero temperature elastic potential energy is

$$U_0 = \frac{h}{4}\sum_{i=1}^{3N} v_i$$

according to basic quantum mechanics.

For each degree of the freedom, we write the partition function as

$$Z^{(i)} = \sum_{n=0}^{\infty} exp(-\frac{\varepsilon_n^{(i)}}{kT}) = \frac{exp(-\frac{x^{(i)}}{2})}{1 - exp(-x^{(i)})}$$

where

$$x^{(i)} \equiv \frac{hv_i}{kT}$$

The vibration entropy for its degree of freedom is

$$S_{vib}^{(i)} = kInZ^{(i)} + kT\frac{\partial InZ^{(i)}}{\partial T} = \frac{kx^{(i)}}{exp(x^{(i)} - 1)} - kIn[1 - exp(-x^{(i)})]$$

where the total vibration entropy is

$$S_{vib} = \sum_{i=1}^{3N} S_{vib}^{(i)}$$

The Gibbs free energy difference of two configurations (a) and (b) can be written as

$$\Delta G = G^{(a)} - G^{(b)}$$
$$= \left[E_0^{(a)} - E_0^{(b)}\right] + \left[U_0^{(a)} - U_0^{(b)}\right] + \left[E_k^{(a)} - E_k^{(b)}\right] + p\left[V^{(a)} - V^{(b)}\right]$$
$$- T\left[S^{(a)} - S^{(b)}\right]$$

We are interested in the case, where the two configurations have the same number of atoms, e.g.,

$$2\mathrm{HfO} \rightarrow \mathrm{Hf} + \mathrm{HfO_2}$$

Since the average kinetic energies are equal at the same temperature, thus in such case one has

$$E_k^{(a)} - E_k^{(b)} = 0$$

Furthermore, at relatively high temperature, the vibration entropy dominates the entropy term, thus we have used S_{vib} instead of S as an approximation. The pV term difference is also neglected. Hence,

$$\Delta G \approx \left[E_0^{(a)} - E_0^{(b)}\right] + \left[U_0^{(a)} - U_0^{(b)}\right] - T\left[S_{vib}^{(a)} - S_{vib}^{(b)}\right]$$

Supplementary Note 3: Surface energy calculations

We calculated the surface energies of tetragonal and monoclinic HfO_2, using various surface configuration. In particular, we selected 5 typical inequivalent surfaces for tetragonal HfO_2, and 11 for monoclinic HfO_2, as listed in Supplementary Table 6. The surface energy σ is defined as

$$\sigma = \lim_{N\to\infty} \frac{1}{2}(E_{slab}^{N} - NE_{bulk}) \tag{1.1}$$

where E_{slab}^{N} represents the total energy of an N-layer slab, E_{bulk} is the total energy of the bulk phase with the number of atoms equaling to one slab layer, and the 1/2 factor accounts for two surfaces. However, it has been pointed out that the bulk energy cannot be simply chosen as that derived from a primitive cell[16]. Rather, in order to avoid the error as N goes to infinity, E_{bulk} can be derived using a curve fitting[17]

$$E_{slab}^{N} \approx 2\sigma + NE_{bulk} \tag{1.2}$$

Here, various N values should be attempted, and E_{slab}^{N} can be plotted as a function against N. The slope will be the appropriate E_{bulk} value to be used for Equation (1.1).

For all slab models, we established symmetric slabs with 1.5 nm vacuum space separating each slab with its periodic images. The central one (if N is odd) or two (if N is even) layers were fixed, while atoms in the other layers were allowed to relax[18]. For any particular surface, we selected more than 3 models with different N values, from which we derived E_{bulk} through curve fitting. As shown in Supplementary Table 6, our results for tetragonal and monoclinic hafnia surface energies are consistent with the published values by Atashi et al.[19] and Luo et al.[20]

Our calculation indicates that the (110) surface of tetragonal hafnia and the $(\bar{1}11)$ surface of monoclinic hafnia are the two with the lowest surface energy. The actual grains cannot only contain the lowest-energy surfaces, because they typically also involve surfaces that are perpendicular (or nearly-perpendicular) to the preferred low-energy surface. We consider 3D grains with six surfaces, where three kinds of surfaces are present. The surface energy effect will be more prominent when the grains are small. Therefore, we estimated the energies of tetragonal and monoclinic hafnia grains with various dimensions. The energy of a grain can be calculated as

$$E_{grain} = E_{supercell} + E_{surface} \tag{1.3}$$

where the supercell energy is derived from primitive cell calculations, and the total surface energy has contributions from all six surfaces. Therefore, we should consider the lowest total surface energy from various surface combinations. According to the data from Supplementary Table 6, we chose (110), $(\bar{1}10)$, and (001) surfaces for tetragonal hafnia, as well as $(\bar{1}11)$, $(1\bar{1}2)$, and $(12\bar{1})$, for monoclinic hafnia (see Supplementary Figure 9-10). The grain energy difference

$$\Delta E_{grain} = E_{grain}^{tetragonal} - E_{grain}^{monoclinic}$$

was estimated, using grains from 12 atoms (Hf_4O_8) to 8748 atoms ($Hf_{2916}O_{5832}$). The relation between ΔE_{grain} and the number of atoms per grain is plotted in Supplementary Figure 11. While the monoclinic grains are energetically favorable at large dimensions,

the tetragonal grains become the preferred configuration when the number of atoms goes below ~4000, or ~3.5 nm side length.

Supplementary Note 4: Oxygen vacancy formation energies in monoclinic and amorphous HfO_2

The oxygen vacancy formation energy was evaluated by calculating a perfect hafnia supercell against the one with one oxygen atom removed. It is defined as

$$E_{form} = E_{defective} - E_{perfect} + \mu_O \tag{1.4}$$

where $E_{defective}$ and $E_{perfect}$ are the total energies of a defective supercell with oxygen vacancy and the perfect stoichiometric supercell, respectively. μ_O is the chemical potential of oxygen, which is here taken as a half of the oxygen molecule energy. We have first calculated a 3×3×3 monoclinic HfO_2 supercell that contains 324 atoms. Vacancies were introduced either on the III-coordination or IV-coordination O sites. The formation energy of the latter (6.26 eV) is lower than the former (6.39 eV) by ~0.13 eV. Subsequently, we built up an amorphous HfO_2 model cell, also with 324 atoms, using a standard melt-and-quench approach. Oxygen vacancies were introduced on five distinct sites, as illustrated in Supplementary Figure 12. The corresponding formation energies are listed in Supplementary Table 7. The formation energy can be as low as 5.58 eV, and even the highest value (6.33 eV) is still comparable to that of monoclinic HfO_2. Considering that oxygen vacancies tend first to emerge on sites with the lowest formation energy, the results show that it is substantially easier to create oxygen vacancies in amorphous HfO_2 than in crystalline (typically monoclinic) HfO_2.

Supplementary Note 5: Advantage of GGA-1/2

On account of the semiconductor band gap problem due to GGA, a proper correction method is required which should be both fast and accurate. As HfO_2 does not belong to the strongly corrected material class, the GGA+U method is not proper. Moreover, hybrid functional calculation or the quasi-particle approach within the GW approximation are both computationally too demanding. Therefore, we adopted the

self-energy corrected GGA-1/2 method[21, 22] for electronic structure calculations for the filament-in-dielectric supercell, which fits normal oxides like HfO_2. The optimum cutoff radius for the O PBE self-energy potential[23] was calculated to be 2.7 bohr in HfO_2, through a variational method. No empirical parameter was involved in the GGA-1/2 calculation. The GGA-1/2 electronic structure for monoclinic HfO_2 is comparable with that of the Heyd-Scuseria-Ernzerhof (HSE06) hybrid functional result (see Supplementary Figure 13).

Supplementary References

1. Adam, J., Rogers, M. The crystal structure of ZrO_2 and HfO_2. Acta. Crystallogr. **12**, 951-951 (1959).
2. Stacy, D. W., Johnstone, J. K., Wilder, D. Axial thermal expansion of HfO_2. J. AM. Ceram. Soc. **55**, 482-483 (1972).
3. Aarik, J. et al. Texture development in nanocrystalline hafnium dioxide thin films grown by atomic layer deposition. J. Cryst. Growth **220**, 105-113 (2000).
4. Foster, A. S., Gejo, F. L., Shluger, A., Nieminen, R. M. Vacancy and interstitial defects in hafnia. Phys. Rev. B **65**, 174117 (2002).
5. Caravaca, M., Casali, R. Ab initio localized basis set study of structural parameters and elastic properties of HfO_2 polymorphs. J. Phys. Condens. Matter. **17**, 5795-5811 (2005).
6. Demkov, A. Investigating alternative gate dielectrics: A theoretical approach. Phys. Status Solidi B **226**, 57-67 (2001).
7. Manory, R. R. et al. Growth and structure control of HfO_{2-x} films with cubic and tetragonal structures obtained by ion beam assisted deposition. J. Vac. Sci. Technol. Vac. Surf. Films **20**, 549-554 (2002).
8. Curtis, C., Doney, L., Johnson, J. Some properties of hafnium oxide, hafnium silicate, calcium hafnate, and hafnium carbide. J. AM. Ceram. Soc. **37**, 458-465 (1954).
9. Zhu, L., Zhou, J., Guo, Z., Sun, Z. Metal−metal bonding stabilized ground state structure of early transition metal monoxide TM−MO (TM = Ti, Hf, V, Ta). J. Phys. Chem. C. **120**, 10009-10014 (2016).
10. Mao, G.-Q. et al. Oxygen migration around the filament region in HfO_x memristors. AIP Adv. **9**, 105007 (2019).
11. Bayarjargal, L. et al. Synthesis of Hf_8O_7, a new binary hafnium oxide, at high pressures and high temperatures. High Press Res. **37**, 147-158 (2017).
12. Dhanunjaya, M. et al. Hafnium oxide nanoparticles fabricated by femtosecond laser ablation in water. Appl. Phys. A **125**, 74 (2019).
13. Xie, Y.-q., Peng, K., Yang, X.-x. Electronic structures and properties of Ti, Zr and Hf metals. J. Cent. South Univ. Technol. **8**, 83-88 (2001).
14. Ahuja, R., Wills, J. M., Johansson, B., Eriksson, O. Crystal structures of Ti, Zr, and Hf under compression: Theory. Phys. Rev. B **48**, 16269 (1993).
15. Zhang, C.-B., Li, W.-D., Zhang, P., Wang, B.-T. First-principles calculations of phase transition, elasticity, phonon spectra, and thermodynamic properties for hafnium. Comput. Mater. Sci. **157**, 121-131 (2019).
16. Boettger, J. Nonconvergence of surface energies obtained from thin-film calculations. Phys. Rev. B **49**, 16798-16800 (1994).

17. Fiorentini, V., Methfessel, M. Extracting convergent surface energies from slab calculations. J. Phys. Condens Matter **8**, 6525-6529 (1996).
18. Marlo, M., Milman, V. Density-functional study of bulk and surface properties of titanium nitride using different exchange-correlation functionals. Phys. Rev. B **62**, 2899-2907 (2000).
19. Mukhopadhyay, A. B., Sanz, J. F., Musgrave, C. B. First-principles calculations of structural and electronic properties of monoclinic hafnia surfaces. Phys. Rev. B **73**, 115330 (2006).
20. Luo, X. et al. Combined experimental and theoretical study of thin hafnia films. Phys. Rev. B **78**, 245314 (2008).
21. Ferreira, L. G., Marques, M., Teles, L. K. Approximation to density functional theory for the calculation of band gaps of semiconductors. Phys. Rev. B **78**, 125116 (2008).
22. Xue, K.-H., Yuan, J.-H., Fonseca, L. R., Miao, X.-S. Improved LDA-1/2 method for band structure calculations in covalent semiconductors. Comput. Mater. Sci. **153**, 493-505 (2018).
23. Yuan, J.-H. et al. GGA-1/2 self-energy correction for accurate band structure calculations: the case of resistive switching oxides. J. Commun. Phys. **2**, 105005 (2018).
